# A Positive Affective Neuroendocrinology Approach to Reward and Behavioral Dysregulation

**DOI:** 10.3389/fpsyt.2015.00093

**Published:** 2015-07-02

**Authors:** Keith M. Welker, June Gruber, Pranjal H. Mehta

**Affiliations:** ^1^Department of Psychology and Neuroscience, University of Colorado Boulder, Boulder, CO, USA; ^2^Department of Psychology, University of Oregon, Eugene, OR, USA

**Keywords:** testosterone, cortisol, emotion, affect, reward, self-regulation, sex differences

## Abstract

Emerging lines of research suggest that both testosterone and maladaptive reward processing can modulate behavioral dysregulation. Yet, to date, no integrative account has been provided that systematically explains neuroendocrine function, dysregulation of reward, and behavioral dysregulation in a unified perspective. This is particularly important given specific neuroendocrine systems are potential mechanisms underlying and giving rise to reward-relevant behaviors. In this review, we propose a forward-thinking approach to study the mechanisms of reward and behavioral dysregulation from a positive affective neuroendocrinology (PANE) perspective. This approach holds that testosterone increases reward processing and motivation, which increase the likelihood of behavioral dysregulation. Additionally, the PANE framework holds that reward processing mediates the effects of testosterone on behavioral dysregulation. We also explore sources of potential sex differences and the roles of age, cortisol, and individual differences within the PANE framework. Finally, we discuss future prospects for research questions and methodology in the emerging field of affective neuroendocrinology.

## Introduction

In recent decades, separate lines of research have investigated the psychological, neural, and neuroendocrine mechanisms of behavioral dysregulation, defined here as appetitive, risky behaviors, such as sexual risk-taking (e.g., unprotected sex), dangerous driving, risky financial decision making, and substance use. Two bodies of research of relevance have independently examined the hormonal mechanisms of behavioral dysregulation. One perspective investigates the hormonal predictors and mechanisms (particularly testosterone), while another has focused on reward dysregulation, defined by researchers as the pursuit of pleasurable feelings and stimuli and heightened responsiveness to positive, reward-related stimuli [e.g., Ref. (1–3)]. As we argue, these systems share overlapping psychological and physiological mechanisms, yet have not been simultaneously deployed to understand behavioral dysregulation. Thus, there is a need to integrate these disparate lines of work into a common theoretical framework. This framework should not only be consistent with extant findings but also make novel predictions to be tested in future research.

In this paper, we propose a forward-thinking approach to study reward motivation and behavioral dysregulation, referred to as the positive affective neuroendocrinology (PANE) approach. The PANE approach incorporates existing research in the hormonal mechanisms of behavioral dysregulation with research on reward dysregulation and related positive affectivity. This approach suggests that reward dysregulation underlies the established links between testosterone and behavioral dysregulation. The PANE approach also holds that testosterone increases reward processing – or neural activity in the reward-relevant regions of the brain – and reward motivation, which in turn increase the likelihood of behavioral dysregulation. More specifically, this framework posits that increases in reward mediate the effects of testosterone on behavioral dysregulation. In this paper, we provide a focused review of the roles of testosterone in modulating behavioral dysregulation and then discuss how reward dysregulation represents a crucial mechanism in this relationship. We also explore the potential sources of sex differences and the effects of age, cortisol, and individual differences within a PANE perspective. Finally, we close with a discussion of future research prospects in the emerging field of affective neuroendocrinology.

## Evidence for PANE

What is the evidence for the PANE framework of reward and behavioral dysregulation? In the following sections, we discuss the evidence from three areas for why the association between testosterone and behavioral dysregulation may be mediated by elevated reward dysregulation: (1) evidence showing that testosterone is a predictor and mechanism of behavioral dysregulation, (2) evidence for how reward dysregulation is a critical mechanism of behavioral dysregulation, and (3) evidence that testosterone increases reward dysregulation.

### Testosterone and behavioral dysregulation

Testosterone, a steroid hormone and end-product of the hypothalamic–pituitary–gonadal (HPG) axis, is of prime relevance to behavioral dysregulation. In men, testosterone is primarily produced in the testes, while women’s testosterone is produced in smaller quantities by the ovaries and adrenal cortex (4). Testosterone also has a diurnal cycle, where testosterone is highest upon waking and decreases across the day, flattening in the afternoon (5). Researchers often distinguish between organizational effects of testosterone – the “permanent modification of brain structure and function during prenatal and early postnatal life due to exposure to testosterone” [Ref. (6), p. 15268] – and activational effects of testosterone – temporary, non-developmental moment-to-moment effects of testosterone that modulate affect, cognition, and behavior upon administration or release of testosterone.

Research on the dysregulatory behavioral effects and correlates of testosterone confirms both stable and dynamic, contextual psychological effects of the HPG axis. Studying the stable, trait-like elements of testosterone involves inferring stable levels of testosterone from either multiple samples at the same time of day [e.g., Ref. (7)], or taking a sample at one time of the day for all participants, after a period of neutral activity (8). Support for this approach comes from reports that testosterone concentrations are relatively stable when measured at the same time of day (9). Thus, baseline testosterone can be considered a trait-like index of testosterone. Large-scale studies have linked baseline testosterone to several dysregulatory behaviors in army veterans, such as substance use, previous juvenile delinquency, and law breaking [e.g., Ref. (10)]. Baseline testosterone is also positively associated with risky financial decision making and preferences [see Ref. (11), for a review; e.g., Ref. (12, 13)].

Collectively, there is a weak positive association between stable, trait-like testosterone concentrations and risk-taking, with some inconsistent findings. For instance, Stanton et al. (14) report a non-linear relationship between testosterone and risk-taking, suggesting that risk-taking is elevated in low and high testosterone individuals, but not those with middle-range testosterone concentrations. Additionally, Sapienza et al. (6) report a positive association between testosterone and risk-taking in women, but not men. Furthermore, Schipper (15) found a negative association between testosterone and risk-aversion for gains, but not losses, in men.

The lack of strong effects of baseline testosterone on risk-taking in humans may be due to the potential for testosterone concentrations to alter in response to social events. Although baseline testosterone may predict how individuals generally respond and act across a wide variety of contexts and self-reported psychological traits, a more fine-tuned assessment of testosterone may be needed for assessing situation-specific behaviors. For example, previous work has examined the behavioral effects of testosterone responses to competitions (16–19), opposite sex interactions (20), men’s interactions with women (21, 22), social exclusion (23), holding dominant vs. submissive postures (24), and aggressive provocation (25). These dynamic effects of testosterone are theorized to be more robustly associated with context-specific social behaviors than baseline testosterone (26), and this notion is supported by several emerging studies [e.g., Ref. (16, 27, 28)] showing robust effects of testosterone changes predicting aggressive behavior in social contexts. This work is also bolstered by a recent study showing that acute changes in testosterone in response to monetary wins and losses also are associated with increased financial risk-taking in men (29). Collectively, these studies suggest that both baseline and dynamic changes in testosterone are positively related to a range of dysregulatory behaviors, particularly risk-taking.

### Reward-seeking and behavioral dysregulation

Theories of behavioral dysregulation (e.g., risk-taking) have distinguished between appetitive, approach-oriented, reward motivations based on achieving satisfaction and avoidance motivations based on reducing or avoiding negative consequences, such as pain, punishment, or losses [e.g., Ref. (30–32)]. Affective and motivational accounts of risk-taking specify reward dysregulation as a critical component [e.g., Ref. (33)]. Additionally, elevations in reward-seeking facilitate the heightened risk-taking behaviors associated with adolescence [see Ref. (34), for review] and underlie a host of dysregulatory behaviors, such as addictive gambling (35), substance abuse (36), traffic violations (37), and childhood obesity (38). In this work, both the elevated experience of positive emotions and the experience of excessive reward motivation are critical components to behavioral dysregulation.

The deleterious effects of reward motivation and excessive positive emotion also emerge in clinical disorders. Disorders associated with risk-taking behavior, such as bipolar disorder (BD), are characterized by elevated and abnormally persistent positive emotions (39), excessive reward pursuit and deficits in reward-related learning [e.g., Ref. (40)], and deficits in positive emotion regulation [e.g., Ref. (41–43)]. BD is often characterized by elevated risk-taking behaviors and impulsivity (44, 45), such as substance use (46), impulsive gambling behavior (47), aggressive behavior (48), and harmful substance use (46). Broadly, deficits in the behavioral approach system (BAS) are thought to characterize BD and elevated behavioral dysregulation (49).

The effects of positive emotion regulation and elevated, persistent positive affect on behavioral dysregulation are becoming increasingly known. When experiencing urgent positive emotions, people are more likely to engage in a variety of dysregulatory behaviors, such as substance use and risky-sexual behavior (50, 51). Elevated reward processing and positive affect have a robust association with risk-taking (52). Additionally, dysregulatory behaviors such as substance use, binge eating, and risky-sexual behavior, are more likely to occur in the context of positive emotions (50, 51). Elevated reward processing and positive affect have a robust association with risk-taking (52). Additionally, elevated reward-sensitivity uniquely characterizes a subpopulation of drug addicts that are motivated toward drug addiction through the presence of potential for rewards (53, 54).

Research on clinical disorders has also provided insights into the fundamental affective mechanisms of behavioral dysregulation. This research suggests that reward dysregulation is a critical component of behavioral dysregulation. Affective accounts of risk-taking specify reward dysregulation as a critical component [e.g., Ref. (33)]. Clinical disorders associated with risk-taking behavior, such as BD, are characterized by elevated and abnormally persistent positive emotions (39), excessive reward pursuits and deficits in reward-related learning [e.g., Ref. (40)], and deficits in positive emotion regulation [e.g., Ref. (3, 41, 42)]. BD is often characterized by elevated risk-taking behaviors and impulsivity (44, 45), such as substance use (46), impulsive gambling behavior (47), aggressive behavior (48), and harmful substance use (46). In addition to excessive positive emotion, this heightened irritability may also potentiate behavioral dysregulation, such as impulsive aggression (55).

#### Reward-Related Neural Function and Behavioral Dysregulation

In addition to elevated positive affect and reward motivation, neural structures related to positive affect and reward also predict behavioral dysregulation. The reward system has been broadly thought to be the neural basis of the BAS, which operates via the mesolimbic dopaminergic network (56, 57). Connectivity between these dopaminergic regions is theorized to form the basis of the neural circuits of reward and appetitive behavior [see Ref. (58) for a review]. Broadly, this reward system of the brain utilizes several key dopamine-linked structures, such as the ventral tegmental area (VTA) and nucleus accumbens (NAcc), the latter of which is nested in the ventral striatum (59–61). Within these regions, the VTA has numerous dopaminergic pathways with output to the hippocampus, amygdala, medial pre-frontal cortex (PFC) ventral pallidum, and of prime relevance, the NAcc [Ref. (62), for a review]. For example, dopaminergic neuron activation in the VTA that stimulates the NAcc aids in reinforcing responses to food and drugs used in substance abuse [see Ref. (63), for a review], as well as reward cues (64). The NAcc also plays a critical role in affect and appetitive motivation, reaction to novel stimuli, reward-related learning, responses to delayed reward, controlling feeding, and hedonic taste preferences [e.g., Ref. (65–70)]. More broadly, dopamine release in the ventral striatum is associated with self-reported euphoria in humans [e.g., Ref. (71)] and is thought to be a critical modulator of reward anticipation in mammals (72).

**Figure 1:**
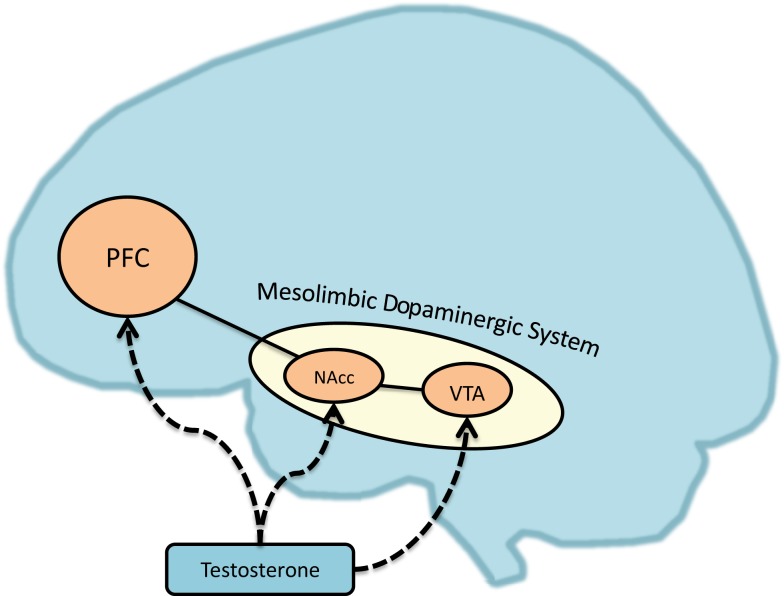
**Testosterone’s effects on brain regions associated with reward dysregulation**. PFC, prefrontal cortex; NAcc, nucleus accumbens; VTA, ventral tegmental area.

Dysregulation in the dopaminergic system has attracted considerable attention in researching behavioral dysregulation and related psychiatric disorders [see Ref. (59, 73), for a review]. For instance, the dopaminergic reward system and dopamine receptor polymorphisms have been linked to the crucial rewarding effects of substance abuse and addiction (74) and pathological gambling [e.g., Ref. (75–77)]. The dopamine system and receptors also modulate increased risky-decision making in humans [e.g., Ref. (78, 79)] and impulsive behavior in rodents [e.g., Ref. (80)]. Furthermore, neural theories of self-regulation (81, 82) hold that self-control is a function of the balance of activation and connectivity between the mesolimbic dopaminergic regions and regions of the PFC – a putative mechanism of self-control, inhibiting craving, and emotional control (74, 83–85). As we will discuss, testosterone modulates activity in these regions (see Figure 1).

### Testosterone and reward

Our evidence for testosterone’s role in reward-seeking comes from three areas of research: testosterone and reward-seeking behavior, testosterone and reward-related affect, and testosterone and the neural circuitry of reward.

#### Testosterone and Reward-Seeking Behavior and Traits

Without involving affective and neural processes, testosterone is associated with increased reward-focused traits, sensation seeking, and impulsive behaviors in humans and animals [e.g., Ref. (86–92)]. Additionally, previous work suggests that exogenous testosterone administration can shift sensitivity from punishment to reward dependency (93). Testosterone changes are also associated with increased monetary gains in stock traders (94). Broadly, this work suggests that testosterone increases motivation to seek rewards.

#### Testosterone and Reward-Related Affect

Testosterone may be related to reward-seeking behaviors, but is it associated with reward-related affect? Work using exogenously administered testosterone suggests that testosterone may shift focus away from withdrawal-related emotions to approach-related, reward-focused aggression (95, 96), and increase subjective and physiological measures of sexual-arousal (97). Testosterone may also be associated with approach-related positive affect. Indeed, testosterone increases are correlated with increased enjoyment of competition in decisive victories (98). Additionally, there is a well-established negative correlation between testosterone and depressive symptoms [see Ref. (99), for a review]. Previous work also suggests that exogenously administered testosterone can also decrease depression (100, 101) and increase manic symptoms (102). Furthermore, in women with BD, testosterone concentrations positively predict the number of manic episodes and suicide attempts (103).

#### Testosterone and Reward-Related Neural Function

Broadly, both testosterone’s organizational and activational effects on the brain are associated with neural regions linked to increased dominance, reward, and approach behaviors [see Ref. (26, 104–106) for reviews; Ref. (107)]. Relevant to the current framework, an expansive literature suggests that testosterone is linked to reward-related neural function, both within animal and human literature. We summarize these associations in Figure 1. Animal research suggests testosterone modulates the dopaminergic system (108–110) and dopamine-linked sexual behaviors in rodents [e.g., Ref. (111, 112)], and has rewarding effects via the mesolimbic dopaminergic system [see Ref. (113), for a review]. For instance, rats show conditioned place preference for regions where they received testosterone injections, and this effect is mediated by dopamine function in the ventral striatum and NAcc (114, 115). Supporting this idea, research in hamsters also suggests testosterone can facilitate dopaminergic activity in the NAcc (116). Furthermore, research with California mice suggests testosterone increases in response to victories facilitate future aggression through the expression of androgen receptors in the ventral striatum (117), potentially through dopaminergic activity.

The association between testosterone and reward-related neural activity parallels that of rodent research. In humans, adolescents’ hormonal changes in puberty have also been theorized to increase appetitive motivation by influencing reward-linked brain structures and dopaminergic pathways (118–122). In humans, testosterone administration increases functional connectivity in neural circuits linked with reduced depression (123). Additionally, exogenous testosterone administrations in humans increase ventral striatal responses to financial reward cues in adolescents and adults receiving monetary rewards (124, 125).

In summary, testosterone may increase reward motivation by acting directly on dopaminergic neural structures in the BAS. However, less work has focused on the effects of testosterone and the BAS beyond dopamine-dependent regions. Although some work suggests, for instance, that testosterone is associated with elevated dorsolateral pre-frontal cortex (DLPFC) activation during an anger control induction (126), other work has not revealed associations between testosterone and the DLPFC during aggressive interactions (127). Future research is needed to extend the specificity of the effects of testosterone beyond reward function to the BAS.

#### Reciprocal Associations Between Reward and Testosterone

Overall, the literature reviewed above suggests that testosterone can increase reward processing and dysregulation. However, there also may be a reciprocal effect of reward on testosterone increases. Reciprocal associations are consistent with existing neuroendocrine theories that posit hormones and behavior reciprocally affect each other through feedback loops [e.g., Ref. (128)]. Broadly, contexts that modulate testosterone responses, such as competitive outcomes and sexually attractive individuals, have rewarding properties. For instance, testosterone responses to competitive victories that facilitate aggressive and risk-taking behavior may occur because winning a competition is an enjoyable experience. Research supporting this possibility suggests a positive association between testosterone responses in winners of competitions and enjoyment of the competition (98). Additionally, the dynamic increases in testosterone following winning a competition and decreases following losing have been thought to facilitate changes in reward-dependent learning (129). However, to fully test this hypothesis, research experimentally manipulates reward in multiple contexts while measuring the effects on testosterone fluctuations is needed.

### Critical moderators

In the following section, we highlight potential critical moderators for within the PANE framework, including cortisol, sex, age, and individual differences linked to reward sensitivity and motivation.

#### Interactive Effects with Cortisol

Within the PANE framework, testosterone may interact with other hormones to predict behavioral dysregulation. Emerging work also suggests that cortisol – a glucocorticoid steroid hormone released as the end-product of the HPG axis – interacts with testosterone to modulate dysregulatory behavior [see Ref. (130), for a review]. From a neurobiological perspective, cortisol downregulates androgen receptors, inhibits HPG activity, and inhibits the effects of testosterone on specific tissues [e.g., Ref. (131–134)]. Additionally, the HPG and HPA axes are thought to have mutually inhibitory effects on each other (135). Therefore, it is possible that cortisol may also moderate the psychological and behavioral effects of testosterone. This notion is supported by psychological literature, finding that when cortisol levels are low, but not high, testosterone levels are positively associated with dominance (136), risk-taking (137), perceived status (138), violent crime (139, 140), and externalizing psychopathology in adolescents (141), although others did not find similar associations (10, 142). Recent research also suggests that acute testosterone changes are positively related to earnings in bargaining contexts when cortisol levels decrease, but not increase (143). In summary, not only do cortisol and testosterone have independent effects on costly behavioral dysregulation but these hormones may also co-regulate risk-taking behavior and impulsive traits. Future research is needed to further investigate the extent to which testosterone and cortisol jointly influence self-control related behaviors (144).

#### Sex Differences in the Psychoneuroendocrinology of Behavioral Dysregulation

A large literature suggests men are more impulsive, punishment insensitive, and sensation seeking than women [see Ref. (145), for a meta-analysis; Ref. (146)], and are on average, more risk-taking (147), although the effect sizes are small (148). Men also typically die earlier than women (149, 150), are more likely to die from violent deaths (151), are more aggressive [e.g., Ref. (152–155)], and are more likely to abuse alcohol (156). Additionally, relative to women, men have more psychopathological traits and disorders linked increased impulsivity (39, 157–160).

Sex differences in testosterone are thought to account for sex differences in risk-taking (6). Work by Sapienza and colleagues indicates that both the organizational functions of testosterone in prenatal development – indexed by the ratio of the second to fourth finger digits (161, 162) – and circulating levels of testosterone account for sex differences in risky-decision making. On the level of prenatal exposure to testosterone, previous work has found physiological indicators of prenatal testosterone exposure can alter children’s social and empathic abilities (163). Although numerous cultural and social factors explain gender differences in behavioral dysregulation, both organizational and activational effects of testosterone likely explain a portion of this variability. It is important, however, not to rule out social and cultural factors facilitating differences in behavioral dysregulation between men and women. Gender roles often guide behavior through social pressures and conformity [see Ref. (164, 165), for reviews]. To explain sex differences in dysregulatory impulsivity and risk-taking, it is necessary to account for not only both the nature and nurture, but the interaction between the two (164–166).

The effects of dynamic changes in testosterone on behavior may be specific to men. For example, Carré et al. (16) find that testosterone reactivity mediates the effect of competitive outcomes on aggressive behavior specifically in men but not women. Although several studies investigating the effects of testosterone reactivity on dysregulatory behaviors have primarily focused on samples of men [e.g., Ref. (23, 28)], future research is needed to establish whether the dynamic effects of testosterone on dysregulatory behavior are sex-specific. This work does not imply, however, that testosterone cannot have behavioral and psychological effects in women. Several testosterone administration studies, for example, have produced behavioral and psychological effects of testosterone in samples of exclusively women [e.g., Ref. (167–170)]. Additionally, previous work has identified interactive effects of testosterone and cortisol in samples of both men and women (136, 137).

Several factors may additionally explain smaller psychological and behavioral effects of testosterone in women. First, animal research suggests that females may have less androgen sensitivity compared to males. Although exogenous androgens can influence sexual mounting behaviors in female hamsters, female hamsters are less responsive to the effects of androgens on neuroendocrine function and sexual behavior than males (171, 172). Females have also been found to have decreased androgen receptor immunoreactivity and density compared to males in several regions of the brain (173). Second, compared men, women produce far less testosterone and have less variability in testosterone. This restricted range reduces the statistical power to detect testosterone’s psychological and behavioral effects (174) and this may hinder the detection of these effects in women. Additionally, the type of methodology used to measure testosterone can have sensitivity at different ranges (175) and may not always be well-suited for measuring the decreased concentrations of testosterone in women.

In summary, testosterone explains both intersex and intrasex variability in dysregulatory behavior. Researchers have several obstacles in measuring testosterone, which hopefully will be curtailed with the advent of greater precision in measurement and the accumulation of more data. Further exploring the role of testosterone in reward dysregulation within men and women would advance the study of the psychological effects of testosterone.

#### Age

Another potential moderator of the PANE framework is age. Post-adolescence aging coincides with decreases in testosterone [e.g., Ref. (176)], decline in neural reward-related function [e.g., Ref. (177)], increased preferences for delayed rewards (178), and decreased risk-taking behavior [e.g., Ref. (147)]. Furthermore, developmental researchers have proposed that pubertal increases in sex hormones including testosterone are linked to elevated risk-taking in adolescents (179, 180). Additionally, research on risk-taking suggest that both male and female adolescents engage in more risk-taking compared to adults [e.g., Ref. (181, 182)], leaving greater potential for testosterone to explain risk-taking in adolescents compared to adults. Thus, it is possible that age may moderate associations in the PANE framework and also modulate differences in the mechanisms of testosterone, reward function, and behavioral dysregulation.

#### Individual Differences

Although work on individual differences moderators of testosterone, reward, and behavioral dysregulation is preliminary, the associations in the PANE framework may be modulated by individual differences. For instance, Norman et al. (183) found that trait anxiety moderates the association between testosterone dynamics and impulsive aggression, while Schultheiss and colleagues (184, 185) report that implicit power motive can modulate testosterone responses to competitive contexts. Additionally, the mechanisms in PANE may also be affected by other individual differences related to reward motivation, such as the behavioral inhibition and activation scales [BIS/BAS; (186)] or regulatory focus (187).

### The PANE framework – a summary

**Figure 2:**
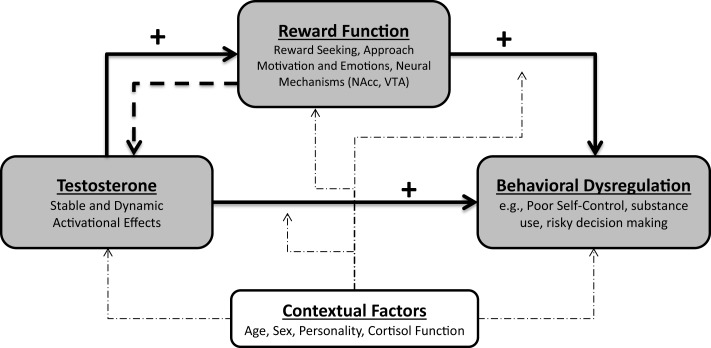
**The PANE framework of reward and behavioral dysregulation**. The PANE framework specifies that elevated stable levels and dynamic increases of testosterone facilitate increased reward function. This increased reward function then facilitates behavioral dysregulation and behaviors indicative of excessive reward pursuit. This perspective also allows for the possibility that reward function can increase testosterone.

The PANE framework provides an organizing framework of existing research showing that the association between testosterone and behavioral dysregulation is mediated by increased reward motivation and reward dysregulation (see Figure 2). Specifically, the PANE approach primarily holds that both stable, trait-like levels and moment-to-moment dynamic changes in testosterone can increase reward dysregulation. Enhanced reward processing – a psychological and neural mechanism of behavioral dysregulation – then increases the likelihood of behavioral dysregulation. Because reward function is affected by testosterone and also serves as a key mechanism of behavioral dysregulation, we argue that reward function is a prime candidate for a mediator of the association between testosterone and behavioral dysregulation. Consistent with contemporary accounts of mediation (188), by specifying reward function as a mediator of the association between testosterone and behavioral dysregulation, we mean that reward function is a causal mechanism in this association. As we review above, testosterone and reward function and motivation modulate a common set of reward-dependent behaviors, and well-established causal directions among testosterone, reward, and behavioral dysregulation suggest that this network of relations is mediated.

The PANE approach is currently at a preliminary state in understanding neuroendocrine function, reward-dysfunction, and behavioral dysregulation. The current paper provides a rationale for why reward function may mediate the association between testosterone and behavioral dysregulation. However, as a whole, this mediation is untested by empirical articles. Future work is needed for researchers to test the overall mediation proposed by the PANE framework. This framework can be measured and tested in numerous forms and contexts, including using both human and animal samples, experimental manipulations of reward, pharmacological testosterone manipulations, testosterone modulating experimental paradigms (e.g., competitive outcomes), and multiple measures of behavioral dysregulation (e.g., poor financial decision making, substance abuse, risky-sexual behaviors). This flexibility allows for the PANE model to be tested and applied by researchers from many backgrounds.

It is necessary to note where the PANE approach differs from other neuroendocrine accounts of behavior. Previous accounts of testosterone and social behavior often indicate testosterone as a biomarker and mechanism of dominance and reproductive behaviors [e.g., Ref. (189, 190)], while recent research has also linked testosterone to threat-based neural function [e.g., Ref. (191)]. It is clear that testosterone modulates these psychological functions in addition to purely reward-related function. However, the literature we review suggests that dominance and sexual behavior are not the only variables regulated by testosterone. As we reviewed, testosterone is related to reward-related neural function, affect, and behaviors, as well as multiple phenotypes of behavioral dysregulation more distal to sexual behavior and dominance, such as substance use, risky-decision making, and sensation seeking. Thus, these behaviors are unlikely to be guided completely by the dominance and sexual behavior-related functions of testosterone. It is possible, instead, that rewards of status-seeking and sexual behavior may actually be a function of the reward-related function of testosterone, but future work is needed to test this possibility. At this point, the PANE framework is designed to be an additive perspective of the effects of testosterone on behavior in addition to other existing accounts.

## Future Directions for Research on Reward Dysregulation, Testosterone, and Behavioral Dysregulation

Although the PANE approach proposes that reward function is a critical mediator in the association of testosterone and dysregulatory behaviors, this research can be developed in several ways. In the following sections, we also propose additional ways the PANE perspective can be expanded: (1) the role of social functioning, (2) translational work in psychiatric populations, (3) integration with neuroendocrine models of aggressive behavior, (4) the positive effects of behavioral dysregulation, and (5) integration with other systems of behavioral dysregulation.

### Decreased social functioning

Testosterone may also increase risk-taking by decreasing social connections with others. It has been long known that socially isolated or disconnected individuals are more likely to engage in reckless behaviors, such as aggression, violence, and drug use (192, 193). This idea is consistent with recent reports suggesting a robust association between having social connections and decreased risk-taking. For instance, having better quality peer and family relationships is associated with decreased risk-taking in adolescents (194, 195) and higher levels of social support are linked with decreased risk-taking in stigmatized sexual ­minorities [e.g., Ref. (196, 197)]. Additionally, self-regulation has been found to be impaired by social exclusion (198). Socially excluded people are also more likely to engage in financial risk-taking (199).

How might testosterone decrease social relationship quality? Broadly, low testosterone is associated with nurturant, pro-social, relationship-promoting behavior (200–202). Basal testosterone is positively associated with having an avoidant, disconnected interpersonal approach, and greater loneliness (203). Testosterone is also positively related to decreased relationship satisfaction and commitment in couples, in both individuals and their romantic partners (204). Additionally, exogenous testosterone can decrease empathy and trust (168, 205), which may impair social relations. Furthermore, the increased risk-taking associated with testosterone function may also in turn decrease relationship quality, further impairing this process.

In summary, by decreasing the quality of social relationships, testosterone may increase the likelihood individuals engage in dysregulatory behaviors, such as maladaptive substance use to cope with poor social relationships [e.g., Ref. (206)]. We additionally suggest this association may be mediated by other processes we previously reviewed. For instance, research suggests having more meaningful family relationships can decrease risk-taking through neural activation indicative of decreased reward sensitivity and increased cognitive control [dorsolateral pre-frontal cortex, Ref. (194)]. Likewise, decreased empathy – an emergent property of reward and positive emotions (207, 208) – may also be related to the association between testosterone and reward processing.

### Translational implications for psychiatric illnesses

Numerous psychological disorders are characterized by trait impulsivity and behavioral dysregulation, such as BD, borderline personality disorder, and attention deficit hyperactivity disorder (39). From the PANE perspective, targeting hormones and affective states leading to behavioral dysregulation presents a novel, translational approach to understanding and treating these disorders. In particular, BD is a prime candidate to investigate the PANE approach. BD is characterized by increased reward sensitivity and difficulties down-regulating reward (2, 41), which may be of particular interest and application for the PANE approach. A chronic, severe, and often fatal psychiatric illness, BD ranks in the top 10 leading causes of worldwide disability by the World Health Organization. The core diagnostic criterion for BD involves periods of abnormally and persistently elevated positive mood (39) and impairments in reward processing have been proposed as a putative endophenotype for BD (209).

Three lines of evidence suggest that BD is a target population for studying testosterone and reward function. First, BD is associated with increased reward sensitivity. For example, people with BD exhibit increased reward reactivity (2, 210, 211), excessive pursuits aimed at obtaining rewards (1, 212), and impairments in reward-related learning (213). Second, empirical models of BD stress the importance of reward dysregulation in the causes and course of the disorder (1, 2, 210–212). Troubles with reward processing persist in BD, even during periods of symptom remission. For example, remitted BD patients report trouble decreasing or down-regulating reward (42), and engage in maladaptive strategies that amplify reward-relevant responses (43, 214), compared with healthy controls. Third, increased reward sensitivity is associated with clinical impairment in BD. Sensitivity to reward predicts increases in manic symptoms over time in BD (215).

Preliminary evidence also suggests that testosterone is important factor for understanding the course and symptom severity in BD. For instance, heightened testosterone levels are associated with significant increases in mania symptoms and severity in BD (103, 216), and oral administration of testosterone has been causally linked to the onset of manic symptoms (102). Future research is needed to understand the hormonal and reward-related mechanisms of BD and other disorders.

### Integration with theories of aggressive behavior

Much of research and theory links aggressive behavior to negative affective systems and threat processing [e.g., Ref. (217–221); see Ref. (26), for a review]. However, the reward systems are also implicated in aggressive behavior [e.g., Ref. (117, 222)]. A model of aggressive behavior accounting for reward and threat-processing may help explain mixed evidence for testosterone and aggressive behavior in neuroendocrine research. Although threat-function and negative affective systems undoubtedly play a critical role in facilitating aggressive behavior, a PANE approach to aggression may help enhance neuroendocrine models of aggressive behavior beyond just accounting for negative affect.

### Exploring the “light side” of behavioral dysregulation

So far, the primary discussion of the PANE approach to impulsive behavioral dysregulation has focused on impulsive behaviors. However, just as calculated, non-impulsive behaviors can have antisocial consequences, not all impulsive acts have negative effects and many can be generous or pro-social to others [e.g., Ref. (223, 224)]. Emerging research suggests testosterone is positively associated with pro-social acts of fairness, cooperation, and reciprocity (205, 225, 226). Because positive emotionality has been found to be linked to pro-social behavior and because neural systems linked to reward are also related to pro-social behavior (227), the PANE approach may also explain the how testosterone can increase pro-social behaviors through positive emotions and reward motivation. Future research is needed to uncover further associations between testosterone, positive emotions, and pro-social behavior.

### Integration with other systems of behavioral dysregulation

Although the PANE perspective specifies reward processing as a central mediator to the association between testosterone and behavioral dysregulation, reward is likely not the only mechanism. For example, one potential mechanism of increased risk-taking and impulsive behavior implicated are the pre-frontal regions of the brain linked to impulse control and self-regulation, such as the orbitofrontal cortex (OFC), which is related to risky-decision making [Ref. (228); see Ref. (179), for a review]. Although the literature suggesting testosterone can modulate the OFC is not as expansive as the testosterone-reward literature, the association testosterone has with aggression and risk-taking has been in part explained by decreased OFC activation (127) and volume in males (179). Furthermore, research suggests testosterone decreases connectivity between the OFC and subcortical areas like the amygdala (229, 230).

The effects of testosterone on the reward and self-control systems fit well with established dual-systems models of self-control. Hofmann et al. (231) specify that two systems modulate self-control: an impulsive associate system that automatically triggers impulsive responses to the environment and a reflective system providing executive control of overriding impulses and implementing strategic plans for goal pursuit. Based on what is known of the neural effects of testosterone, testosterone changes may modulate the activation of both impulsive and reflective systems. As more research emerges, one broad goal of the PANE perspective and surrounding research will be to integrate more with other mechanisms and perspectives of behavioral dysregulation, such as the dual-systems approach.

## Conclusion

The PANE perspective is designed to organize the work on testosterone, reward dysregulation, and behavioral dysregulation into one coherent framework to stimulate research on behavioral dysregulation. The endocrine mechanisms discussed in this paper may also influence behavioral dysregulation through other mechanisms than reward [such as self-control systems and the OFC, Ref. (127)]. However, the evidence is clear that reward dysregulation is a principal mechanism modulating dysregulatory behaviors and it is necessary to unify this work into a larger framework. Broadly, researchers need to identify mediating psychological and neural mechanisms for the association between testosterone and behavioral dysregulation, and to unify these processes in an elegant, unified model. Together, both neuroendocrine and reward motivation accounts of behavioral dysregulation may hold promise in explaining poor self-control and impulsive behaviors across a wide range of clinical, health, and social contexts.

## Conflict of Interest Statement

The authors declare that the research was conducted in the absence of any commercial or financial relationships that could be construed as a potential conflict of interest.
